# P30 Impact of introducing antiviral stewardship reviews in a major acute teaching hospital

**DOI:** 10.1093/jacamr/dlaf230.037

**Published:** 2025-12-04

**Authors:** S W Chong, S K Zaidi, T Whitfield

**Affiliations:** Salford Royal Hospital, Northern Care Alliance (NCA) NHS Foundation Trust, Salford, UK; Salford Royal Hospital, Northern Care Alliance (NCA) NHS Foundation Trust, Salford, UK; Salford Royal Hospital, Northern Care Alliance (NCA) NHS Foundation Trust, Salford, UK

## Abstract

**Background:**

All hospital trusts are required to have stewardship programmes to promote the judicious use of antimicrobials. These programmes have historically focused on antibiotics, although attention to antifungals has increased. However, few studies in the literature focused on antiviral stewardship (AVS). A shortage of parenteral aciclovir (ACV) arose in May 2024. During the contingency planning phase, we read of the success of virology stewardship reviews at University Hospitals Birmingham^1^ and decided to introduce this locally.

**Objectives:**

To introduce virology stewardship reviews at Salford Royal Hospital.; to reduce unnecessary usage of parenteral aciclovir ;and to promote good antiviral stewardship practice.

**Methods:**

Virtual AVS reviews were performed by consultant virologist(s) and an antimicrobial pharmacist. Patients prescribed ACV IV were identified via the electronic prescribing system. Reviews were conducted biweekly initially then weekly for the last 2 weeks. Data was collected over 12 weeks between 9/5 and 31/7/24.

**Results:**

A total of 41 reviews were performed for 19 patients The average time taken was 20 min per session. AVS recommendations were: continue (29), stop (6), dose modification (5) and oral switch (1). During the intervention period, mean monthly consumption of ACV IV in defined daily doses (DDDs) fell to 35.5 compared to 61.7 over the same period the previous year. Total expenditure on ACV IV over May–July 2024 was £950, a saving of £1299 compared to May–July 2023. Other additional findings were as follows. (i) ACV continued whilst awaiting cerebrospinal fluid (CSF) viral PCR results from an external laboratory with turnaround times of >48 h. In some cases, ACV could have been stopped in the context of clinical review, timing of CSF collection and brain imaging. The average time taken for PCR result to be received and actioned during the study was 8.3 days, which is a significant delay that is being addressed. (ii) ACV was prescribed unnecessarily for suspected viral meningitis in immunocompetent patients in some cases. There are no proven benefits of continuing ACV IV in most causes of viral meningitis.^2^ With regular reinforcement we found that this practice reduced over the timeframe of the intervention. (iii) Dosing weights for ACV were inconsistent between actual, ideal and adjusted body weight. British National Formulary recommends ideal body weight whereas other references^3^ recommend adjusted body weight. This was taken to the Trust antibiotic steering group for discussion.

**Conclusions:**

Antiviral stewardship reviews reduced consumption of IV aciclovir. Regular reviews and feedback to clinicians helped optimize treatment plans. Overall, AVS reviews successfully promoted judicious use of ACV and preserved supplies for the highest risk patients.
